# Discovery of Noncancer Drug Effects on Survival in Electronic Health Records of Patients With Cancer: A New Paradigm for Drug Repurposing

**DOI:** 10.1200/CCI.19.00001

**Published:** 2019-05-29

**Authors:** Yonghui Wu, Jeremy L. Warner, Liwei Wang, Min Jiang, Jun Xu, Qingxia Chen, Hui Nian, Qi Dai, Xianglin Du, Ping Yang, Joshua C. Denny, Hongfang Liu, Hua Xu

**Affiliations:** ^1^ The University of Texas Health Science Center at Houston, Houston, TX; ^2^ University of Florida, Gainesville, FL; ^3^ Vanderbilt University Medical Center, Nashville, TN; ^4^ Mayo Clinic, Rochester, MN; ^5^ Department of Veterans Affairs, Tennessee Valley Healthcare System, Nashville, TN

## Abstract

**PURPOSE:**

Drug development is becoming increasingly expensive and time consuming. Drug repurposing is one potential solution to accelerate drug discovery. However, limited research exists on the use of electronic health record (EHR) data for drug repurposing, and most published studies have been conducted in a hypothesis-driven manner that requires a predefined hypothesis about drugs and new indications. Whether EHRs can be used to detect drug repurposing signals is not clear. We want to demonstrate the feasibility of mining large, longitudinal EHRs for drug repurposing by detecting candidate noncancer drugs that can potentially be used for the treatment of cancer.

**PATIENTS AND METHODS:**

By linking cancer registry data to EHRs, we identified 43,310 patients with cancer treated at Vanderbilt University Medical Center (VUMC) and 98,366 treated at the Mayo Clinic. We assessed the effect of 146 noncancer drugs on cancer survival using VUMC EHR data and sought to replicate significant associations (false discovery rate < .1) using the identical approach with Mayo Clinic EHR data. To evaluate replicated signals further, we reviewed the biomedical literature and clinical trials on cancers for corroborating evidence.

**RESULTS:**

We identified 22 drugs from six drug classes (statins, proton pump inhibitors, angiotensin-converting enzyme inhibitors, β-blockers, nonsteroidal anti-inflammatory drugs, and α-1 blockers) associated with improved overall cancer survival (false discovery rate < .1) from VUMC; nine of the 22 drug associations were replicated at the Mayo Clinic. Literature and cancer clinical trial evaluations also showed very strong evidence to support the repurposing signals from EHRs.

**CONCLUSION:**

Mining of EHRs for drug exposure–mediated survival signals is feasible and identifies potential candidates for antineoplastic repurposing. This study sets up a new model of mining EHRs for drug repurposing signals.

## INTRODUCTION

Cancer drug development is increasingly expensive and time consuming. The development of a new drug is estimated to cost $648 million^1^ to $2.5 billion^2^ and takes an average of 9 to 12 years before market availability.^3^ The drug development success rate is less than 8% because of lack of efficacy, excess toxicity, declining research and development, cost of commercialization, and payer influence.^4^ Cancer drugs are now the top sellers among all Food and Drug Administration–approved therapies.^5^ Although many new cancer therapeutics are in development, new methods to accelerate drug discovery are needed. Drug repurposing has received great attention^6,7^ in recent years as one potential solution. A recent study reported that the discovery of new indications of existing drugs accounts for 20% of new drug products.^8^

Electronic health records (EHRs) could be an important source for drug repurposing discovery, but EHRs, which are now present in 96% of health care systems,^9^ have not been extensively leveraged for drug repurposing studies. Recent studies have demonstrated that EHR data can be used as an efficient, low-cost resource to validate drug repurposing signals detected from other sources.^10,11^ Currently, limited research exists on using EHR data for drug repurposing, and most published studies have been conducted in a manner that requires predefined hypotheses. For example, recent evidence has suggested that metformin improves cancer survival^12,13^ and decreases cancer risk in patients with diabetes,^14^ which suggests clinical promise as an antineoplastic agent. We previously found in a retrospective EHR-based study that metformin is associated with superior cancer-specific survival.^10^ This hypothesis-driven method highly depends on domain experts to generate hypotheses and select variables.

In the current study, we take a data-driven approach to detect potential drug repurposing signals using EHR data, with the specific goal of identifying new cancer treatment signals. We evaluated 146 drugs in the Vanderbilt University Medical Center (VUMC) EHR that typically are taken long term for noncancerous conditions and assessed their effects on survival in patients with cancer. We then evaluated signals detected at VUMC by replicating significant associations using the Mayo Clinic’s EHR, searching the biomedical literature for corroborating evidence, and checking cancer clinical trials for support.

## PATIENTS AND METHODS

### Primary Data Source

We used the synthetic derivative (SD),^15^ which is a deidentified copy of VUMC’s EHR. The SD contains comprehensive clinical data for more than 2.3 million patients, including billing codes, laboratory values, pathology/radiology reports, medication orders, and clinical notes. In addition, the SD contains data from the Vanderbilt Cancer Registry, which is maintained by certified tumor registrars according to the standards set forth by the state of Tennessee and the Commission on Cancer.

### Patient With Cancer Definition

This study used patients with cancer identified by the Vanderbilt Cancer Registry, which operates under the mandate of the Tennessee Cancer Registry and the Commission on Cancer. Patients were identified through automated parsing of pathology reports and billing codes.

### Identification of Candidate Drugs for the Study

In the SD, medication information is extracted from both structured (eg, electronic physician orders) and unstructured (ie, clinical notes) data using MedEx.^16^ MedEx has proven high performance on extracting medication names and their signature information in clinical notes.^16^ Here, we required that a drug name must be followed by at least a dosage instruction to account for a prescription to a patient. We have previously shown that the requirement that a drug name be followed by a dosage instruction led to a very high positive predictive value.^10^ To generate a candidate list, we followed two steps. First, we selected normalized drugs used by more than 5,000 individuals, which resulted in 301 candidates, and second, two physicians (J.L.W., J.C.D.) manually reviewed these to exclude known antineoplastics, drugs used in the supportive care of cancer (eg, opiates), over-the-counter drugs, and drugs with short-term indications (eg, antibiotics). Subsequently, 146 candidate drugs remained (see the Data Supplement for the full list). With the assumption that patients were followed for 5 years and the median survival time of the control group was 5 years, with a total of 2.3 million patients and 5,000 who received the drug of interest, we have 89% power to detect a true hazard ratio (HR) of 1.1 (a reduction of 6 months in median survival time assuming exponential distribution) with 5% detection of approximately 150 drugs and a false discovery rate (FDR)–adjusted *P* = .1. For each of the 146 candidate drugs, we developed a multivariable Cox proportional hazards regression model to assess its effect on cancer survival. All other drugs (including noncandidate drugs) that were used by more than 5,000 patients were adjusted as covariates in the multivariable Cox model.

### Study Design, Covariates, and Statistical Analysis

For each drug, we conducted a retrospective cohort study with two comparison groups: an exposure group that comprised patients with one or more prescriptions of the drug in their EHR and a nonexposure group that comprised patients with no prescription of the drug in their EHR. Prescription of a medication was determined by combining both structured electronic physician orders and unstructured clinical notes. Cox proportional hazards regression modeling was used to assess the association of drug exposure with overall survival (ie, time from cancer diagnosis to death) or last medical record date in the EHR (censored). Study covariates were patient demographics (age, biologic sex, race); tumor information (type, stage); diseases with International Classification of Diseases, Ninth Revision, Clinical Modification, codes grouped into phenome-wide association study^17^ phenotypes; and normalized drugs. Because the dimensionality of covariates was high, we conducted variable screening using a univariable Cox model for each disease-related covariate and kept those with *P* < .3. Other variables were directly used without any filtering. We assessed mortality using a multivariable Cox proportional hazards regression model that adjusted for all the selected covariates and reported the *P* values, HRs, and 95% CIs. We used a cutoff FDR-adjusted^18^ *P* < .1 to select the top-ranked drugs associated with cancer survival; this cutoff was chosen to minimize the risk of excessive false negatives at this hypothesis-generating stage.^19^ All analyses were conducted using R 3.1 with the survival, Hmisc, and rms packages (http://www.r-project.org).

### Evaluation

We undertook several experiments to validate the detected signals.

#### Replication using another large site.

Using the EHR and cancer registry at the Mayo Clinic, we replicated the study by following the same design and statistical analysis plan used for the VUMC EHRs. Drugs with a survival signal detected in both institutions’ EHRs also were examined.

#### Search of biomedical literature for supporting evidence.

For additional examination, we identified English-language original publications from PubMed by searching for the drug name plus the term cancer survival. If there was no result or the number of publications was fewer than 10, we also included publications identified by searching the drug name with only the term cancer. We reviewed the abstracts of 100%, 20%, or 10% if the total number of publications was fewer than 20, 21 to 200, or more than 200, respectively. If necessary, the body of available publications also was reviewed. After review, each publication was labeled as one of three categories: evidence to support an antineoplastic effect wherein the drug, alone or in combination, has a cytotoxic effect on cancer cells in vitro or in vivo; evidence to support a carcinogenic effect wherein the drug, alone or in combination, has a proliferative effect on cancer cells in vitro or in vivo; and inconclusive wherein no conclusion can be made about the drug’s cytotoxic or proliferative effect in vitro or in nonrandomized in vivo studies, or the drug failed to demonstrate statistical superiority in a randomized in vivo trial.

#### Search of human interventional cancer trials for supporting evidence.

In a previous study, 25,530 cancer treatment trials were collected from ClinicalTrials.gov.^20^ Among them, we identified 1,068 cancer trials associated with the 146 noncancer drugs used in this study. This subset was manually reviewed and categorized as follows: Category A, the intended primary outcome is survival or a surrogate of survival, including direct effects on a tumor (eg, changes in proliferation indices), solely from the candidate drug (primary effect); category B, the intended primary outcome is survival or surrogate of survival (as in category A) on the basis of synergy between the candidate drug and one or more known antineoplastics (additive effect, including radiotherapy given with the candidate drug); category C, the candidate drug is being used for supportive care purposes or to counter adverse effects of other interventions; and category D, false positives. Of the trials identified as category A or B, we also required that the study be in patients with a current or former diagnosis of cancer; chemoprevention trials were excluded. We also tested whether our signal detection method is significantly different from random selection of drug candidates by using permutation analysis. Additional details are available in the Data Supplement.

## RESULTS

### Drug Repurposing Signals Detected From the VUMC EHR

At VUMC, we identified 43,310 patients with cancer diagnosed at age 18 years or older between January 1, 1995, and December 31, 2010. Patients were a median age of 57 years at diagnosis, 57% were male, and 93% were white. The major cancer types were prostate (5,673; approximately 13%), breast (3,968; approximately 9%), lung (3,346; approximately 8%), and colorectal (2,537; approximately 6%). We collected 2,630 variables for each individual, including three patient demographics (age, biologic sex, race), two tumor information (type, stage), 1,279 diagnoses, and 1,346 medications. We assessed 146 noncancer drugs and detected 30 significantly associated with survival (FDR-adjusted *P* < .1), of which 22 were significantly associated with improved cancer survival. Table 1 lists these 22 drugs, which include statins (rosuvastatin, simvastatin, atorvastatin), proton pump inhibitors (omeprazole, esomeprazole, lansoprazole), angiotensin-converting enzyme inhibitors (ramipril, lisinopril), β-blockers (metoprolol, carvedilol), nonsteroidal anti-inflammatory drugs (NSAIDs; diclofenac, celecoxib), α-1 blockers (tamsulosin), and several others.

**TABLE 1.**
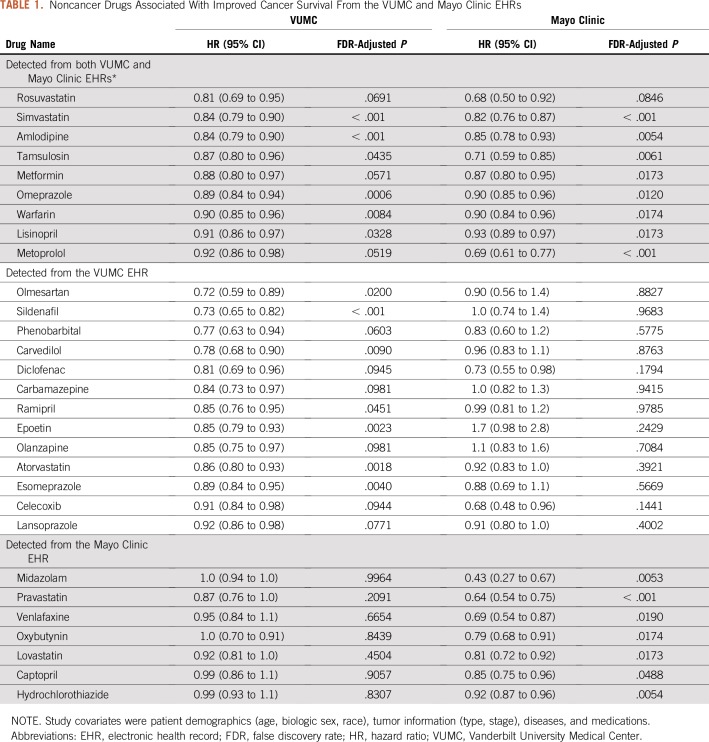
Noncancer Drugs Associated With Improved Cancer Survival From the VUMC and Mayo Clinic EHRs

### Replication Using the Mayo Clinic EHR

**FIG 1.**
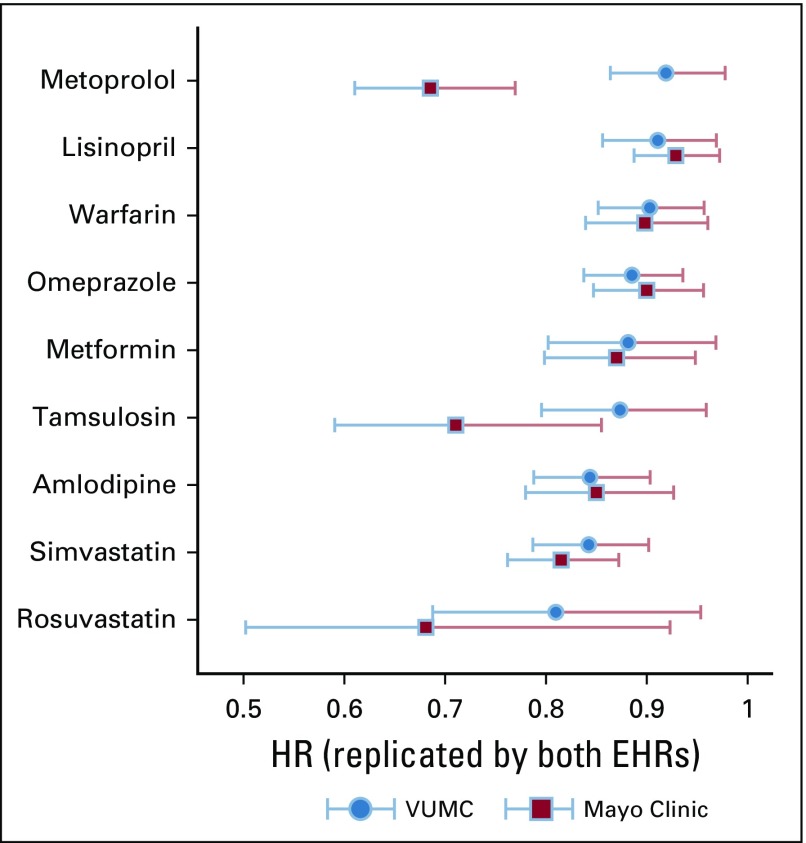
Comparison of the nine drugs detected from the Vanderbilt University Medical Center (VUMC) and replicated by the Mayo Clinic electronic health records (EHRs) by hazard ratio (HR). Study covariates were patient demographics (age, biologic sex, race), tumor information (type, stage), diseases, and medications.

We then sought to replicate the study using 98,366 individual patients diagnosed with cancer at the Mayo Clinic between January 1, 1995, and December 31, 2010. Patients were a median age of 64 years, 57% were male, and 88% were white. The major cancer types were prostate (19,951; approximately 20%), breast (10,415; approximately 10%), lung (9,948; approximately 10%), and colorectal (6,829; approximately 7%). We collected 5,725 variables for each individual, including 1,279 diagnoses and 4,441 medications. Using the same approach, we identified 16 drugs significantly associated with improved survival (Table 1). Among the 22 initially detected drugs from the VUMC EHR, nine were replicated (Table 1). Figure 1 compares the HRs and 95% CIs for the nine replicated drugs. The Data Supplement shows the unadjusted Kaplan-Meier survival curves and associated 95% CIs for the nine drugs detected from both EHRs.

### Validation Using Biomedical Literature

For each of the nine potential drugs detected from VUMC and found in the Mayo Clinic analysis, we searched PubMed for corroborating evidence. A total of 1,348 relevant biomedical publications were found for all nine drugs. As listed in Table 2, all nine drugs have at least one publication that supported an antineoplastic effect, whereas five of them have at least one publication that reported a carcinogenic effect. For all nine drugs, there are more publications that supported their antineoplastic effect compared with their carcinogenic effect. Two drugs, simvastatin and metformin, have a substantial number of publications (20 and 57, respectively). Eighteen of 20 publications supported simvastatin’s antineoplastic effect. Similarly, 40 of 57 publications supported metformin’s antineoplastic effect.

**TABLE 2.**
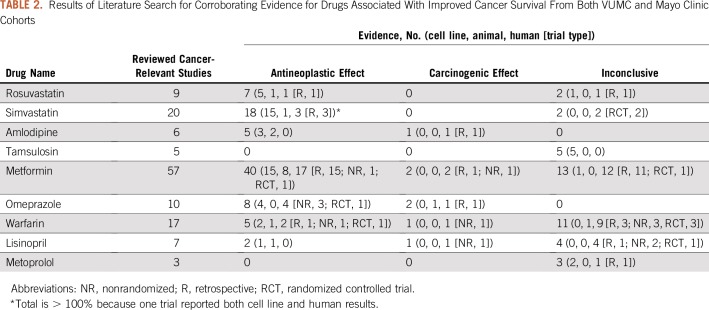
Results of Literature Search for Corroborating Evidence for Drugs Associated With Improved Cancer Survival From Both VUMC and Mayo Clinic Cohorts

### Validation Using Clinical Trials

Manual review of 1,068 candidate trials identified 321 cancer efficacy trials, of which 105 (33%) explored the primary efficacy of the candidate drug (category A) and 216 (67%) explored additive efficacy to known antineoplastic drugs (category B). Of the 146 drugs in this study, 40 (27%) were tested in one or more trials, 28 (19%) were tested in two or more trials, and 17 (12%) were tested in five or more trials. Among the nine drugs with a survival signal replicated across the two clinical sites, four were identified as having completed or ongoing clinical trials (metformin, omeprazole, rosuvastatin, simvastatin). Two of these were among the most heavily studied (metformin and simvastatin, with 64 and 23 trials, respectively). In total, studies that involve the replicated drugs accounted for 30% (95 of 321) of the identified clinical trials.

We conducted permutation analysis^21^ to compare the proposed method with random sampling. The 40 drugs that were tested by at least one cancer efficacy trial served as ground truth (see definition in Data Supplement). Among the 22 drug repurposing signals from VUMC, nine are in ground truth (precision, 41%; recall, 23%). Our method of detecting drug signals outperformed random sampling with *P* = .04 on 100,000 permutations. Detailed results are provided in the Data Supplement.

## DISCUSSION

In this study, we mined large-scale EHR data to detect drug repurposing signals with potential cancer treatment implications. We found strong associations with improved overall cancer survival for statins, proton pump inhibitors, angiotensin-converting enzyme inhibitors, β-blockers, NSAIDs, and α-1 blockers in two EHR systems. We also found evidence for these effects in the biomedical literature and clinical trials. Manual review of the biomedical literature and permutation analysis of cancer clinical trials also show that our proposed method generates potentially valid drug repurposing signals. These findings indicate that the use of EHRs is feasible as a new resource for drug repurposing signal detection. We believe that this study will set up a new model for drug repurposing signal detection using EHRs and thus complement existing methods for drug repurposing studies. For example, scientists have developed computational methods to detect new treatment signals for existing drugs, including structure-based screening,^22,23^ adverse effect networks analysis,^24,25^ genomic and gene expression analysis,^26,27^ and biomedical literature mining.^28,29^ Various data sources from genomics, drug chemical structure,^30,31^ and phenotypic information^24,25,32^ have been explored.

This study is different from previous EHR-based drug repurposing studies.^10,11^ Most previous studies were conducted with a predefined hypothesis about drug and indication. These approaches highly depend on domain experts to define hypotheses and select relevant variables, which could be time consuming if we examine a large number of drugs. In this study, we have taken a data-driven approach that aimed to generate hypotheses. Instead of limited variables defined by domain experts, we included all available information (eg, patient demographics, diseases, drugs) as variables in the analysis. Of course, some important variables likely are not recorded in EHRs and thus not included in the analysis. For example, sociobehavioral determinants of health, including healthy behaviors, are rarely recorded in the current generation of EHRs.^33^

Some noncancer drugs identified in our study have strong evidence for cancer treatment from studies using other data sources. For example, many recent retrospective studies reported metformin associations with improved cancer survival,^12,13^ and a chemoprevention trial in colorectal adenoma was positive.^34^ We identified 64 ongoing or completed clinical trials studying metformin alone or in combination, whose anticancer effect could be related to mammalian target of rapamycin inhibition.^35,36^ Ongoing cancer trials also are evaluating statins for cancer treatment (eg, a trial to assess the efficacy of simvastatin and capecitabine in locally advanced rectal cancer [ClinicalTrials.gov identifier: NCT02161822]). Recent studies have reported that NSAIDs reduce the risk of a wide range of cancers (colon cancer,^37^ oral cancer,^38^ breast cancer,^39^ melanoma^40^) through blocking cellular proliferation and by promoting apoptosis.^37^ Of note, celecoxib (an NSAID) was identified as being the most heavily studied, with 92 ongoing or completed clinical trials,^41^ but the signal for improved survival at VUMC did not replicate at the Mayo Clinic.

Repurposing signals have been found in population-based cohort studies, such as the signal for increased cancer survival in patients who take statins.^42-44^ A smaller number of prospective repurposing trials have reported successes, such as a randomized trial of estradiol therapy of hormone receptor–positive, aromatase inhibitor-resistant advanced breast cancer^45^; a phase II study of pioglitazone in patients with stage IA to IIIA non–small-cell lung cancer (ClinicalTrials.gov identifier: NCT01342770); and an n-of-1 trial that combined metformin with trametinib in a patient with advanced ovarian cancer.^46^ Although some repurposing trials, such as pravastatin added to standard chemotherapy for small-cell lung cancer, have been negative,^47^ increasingly granular phenotyping efforts will lead to refined patient selection. In particular, the advent of routine germline sequencing, somatic tumor profiling, and immunophenotyping will allow for precise patient selection, as in the NCI-MATCH (National Cancer Institute Molecular Analysis for Therapy Choice) trial.^48^ Currently, some drugs have no evidence, or sometimes conflicting findings, about their effects on treating cancer according to existing literature. For example, one study examined captopril and found no clear association between the use of antihypertensive drugs and prostate cancer.^49^ However, another study that focused on users of captopril showed a lower risk of subsequent prostate cancer.^50^ Our literature review was based on a sampling strategy and may have overlooked human trials with strong evidence for antineoplastic effects. Of note, given that this is a repurposing study for candidate drugs not clearly known to have antineoplastic properties, much of the discovered literature was based on cell lines or was retrospective in nature. In addition, the well-known bias to selectively report positive results likely extends to a bias toward reporting antineoplastic results (eg, approximately 350,000 results were found using the medical subject headings term, antineoplastic agents, and only approximately 47,500 for the term carcinogens), which may have affected our findings. Five drugs, including amlodipine,^51^ tamsulosin,^52^ metformin,^53^ warfarin,^54^ and lisinopril^55^ have published results that report an increased risk of cancer. These unsupported signals could be either false positive or novel findings. Additional research with more careful study designs or in-depth mechanism experiments is required to validate or reject these hypotheses.

This study has limitations. Similar to other epidemiologic studies using observational data, our study may suffer from incomplete information and/or unmeasured confounder effects. It is possible, although unlikely because of the time frame of the analysis, that certain clinicians were aware of the potential anticancer effects of some of the study drugs and were intentionally prescribing them for cancer treatment; temporal resolution of self-administered drug exposures is a difficult and as-yet unsolved problem in clinical data extraction.^56,57^ To accommodate the large-scale analysis, our study design is relatively simple: Comparison groups were defined on the basis of mentions of the study drug only without considering the actual exposure details (timing of the drug exposure and drug doses administered) and other potential bias; overall survival, not cancer-specific survival, was used because there were no cancer-specific survival data. However, because the goal of this study was to generate hypotheses, we expect that more carefully designed studies would evaluate such findings in functional models and/or randomized controlled trials. Furthermore, the survival model only examined each drug without considering the combinations of variables. Therefore, our method cannot be used to identify the effect of combinations of drugs.
